# Experimental Studies on Fretting Wear Behavior of PVDF Piezoelectric Thin Films

**DOI:** 10.3390/ma14040734

**Published:** 2021-02-04

**Authors:** Yuanjie Shu, Liaoliang Ke, Jie Su, Fei Shen

**Affiliations:** 1 Institute of Engineering Mechanics, Beijing Jiaotong University, Beijing 100044, China; 17115274@bjtu.edu.cn; 2 School of Mechanical Engineering, Tianjin University, Tianjin 300350, China; jiesu@tju.edu.cn (J.S.); shenfei@tju.edu.cn (F.S.)

**Keywords:** PVDF thin film, fretting wear, applied voltage, friction logs, morphology, wear volume

## Abstract

This paper discusses an in-depth experimental study on the fretting wear behavior of PVDF (polyvinylidene fluoride) piezoelectric thin film against a Si_3_N_4_ ceramic sphere under air conditions. A fretting wear device with a ball-on-plate contact configuration was applied. The changes of displacement amplitude, normal force, and applied voltage were taken into account. The friction logs were used to determine the contact state of the PVDF thin film during the fretting test. The 3D topography instrument and scanning electron microscope (SEM) were used to measure the details of the surface morphology and wear volume. The test results of PVDF thin films under different normal force, displacement amplitude, and applied voltage are summarized through the collection and analysis of experimental data. It is shown that the creep and plastic deformation lead to obvious winkles at the contact surface, which may decrease the specific wear rate of PVDF thin films.

## 1. Introduction

Piezoelectric materials are one of the most important intelligent materials. They exhibit an electric response when stress is applied (direct piezoelectric effect) and conversely tend to deform under an applied electric field (convers piezoelectric effect). Usually, piezoelectric materials are divided into three categories: inorganic piezoelectric materials, piezoelectric polymers, and piezoelectric composites. In the last two decades, the development of piezoelectric materials has received extensive attention owing to their high sensitivity, great electrical performance, and high stability [1].

As important functional materials, piezoelectric thin films provide a good electromechanical coupling and have attracted a lot of attention in the modern industry. Common piezoelectric thin films mainly include lead zirconate titanate (PZT)-based ferroelectric films, lead-free piezoelectric thin films, and piezopolymer thin films [2,3]. The piezopolymer thin films possess outstanding properties, such as excellent mechanical strength, intrinsic piezoelectric capabilities, high flexibility, and great chemical stability, which make them widely used in sensors [4], generators [5,6], actuators [7,8], and transducers [9].

PVDF (polyvinylidene fluoride) thin film is a kind of important piezoelectric thin film, and is widely used in many smart devices. Since these devices are often used in the vibrational environment, fretting wear will inevitably occur at the contact surface of PVDF thin films, and even lead to the failure of devices [10,11]. Fretting wear usually occurs as a relative oscillatory movement between contacting surfaces with a low amplitude of the micron scale. Thus, this phenomenon may arise in any assembly where there exists a vibration source [12,13,14,15]. According to the different direction of relative movements, fretting can be classified into four basic modes: tangential fretting, rotational fretting, radial fretting, and torsional fretting [12]. Previous investigations showed that owing to the impact of external vibration, fretting wear could cause mass decrease and initiate fatigue cracks on the contact pair. More seriously, wear can further lead to the loss of mating of the contact surface, crack growth, and the reduction of fatigue life [16,17,18,19].

In recent years, many researchers have worked on fretting problems, including experimental and theoretical studies. Vingsbo and Söderberg [20] presented an experimental observation and theoretical analysis on the fretting contact region. They mentioned that there were three regions in fretting, i.e., the partial slip region, the mixed region, and the gross slip region. Zhou, et al. [21] presented a map of the variation of tangential displacement vs. tangential force vs. number of cycles for the representation of the contact region. Ding et al. [22] showed that the partial slip region was always associated with slight wear, the mixed region formed cracks, and the gross slip region mainly produced wear and damage of materials. A lot of studies showed that there were many influencing factors on the fretting wear behavior of materials, including frequency [23,24], normal force [25,26], displacement amplitude [27,28], and even temperature [29,30], surface roughness and so on [31,32,33,34,35].

Therefore, it seems necessary to achieve an in-depth understanding on the wear behavior of piezoelectric thin films for their practical applications. However, in the past few decades, the study on the piezoelectric thin film was mainly focused on the performance of polarization and dielectric properties related to its applications [36,37]. Only few works mentioned the friction and wear characteristics of PVDF thin films. Anupama et al. [38] carried out an experimental study on mechanical and wear behavior of pristine PVDF with nanocomposites for the purpose of improving properties of materials. Higham et al. [39] conducted a set of experiments to understand the influence of polymer properties on the fretting wear of the metal surface. It should be pointed out that the wear and fretting contact behaviors of piezoceramics were also concerned by many investigators. Su et al. [40,41] presented a series of theoretic studies on two-dimensional fretting contact and axisymmetric partial slip contact of piezoceramics. They provided an in-depth discussion about the effects of the friction coefficient and punch radius on the fretting contact pressure, tangential traction, in-plane stress, and in-plane electric displacement during different loading phases. Zhou et al. [42] presented a theoretical model for solving the frictional sliding contact problem on monoclinic piezoelectric materials. Chung et al. [43] studied the friction and wear characteristics of PZT thin films by using atomic force microscopy.

In this paper, we performed an in-depth experimental study on the fretting wear of PVDF thin films. The different displacement amplitude and normal force were the main variables in a series of fretting tests. The fretting test data are recorded by the instrument for plotting the friction logs, and the contact surface morphology of PVDF thin films is observed by the 3D optical profiler and scanning electron microscope (SEM). The influences of various normal force, displacement amplitude, and applied voltage are discussed through the collection and analysis of experimental data.

The new aspects of the present paper include the following points: (i) It is the first work to investigate the fretting wear behavior of piezoelectric thin films. We found that the creep and plastic deformation lead to obvious winkles at the contact surface, which may decrease the specific wear rate of PVDF thin films. (ii) Based on the fretting wear test setup, we built a new module with the effect of the voltage. This is also the first time that the effect of the voltage on the fretting wear behavior of piezoelectric materials has been considered. With the increase in voltage, both wear volume and specific wear rate first increase and then decrease. (iii) The fretting wear mechanism is also discussed in detail. The damage type and possible causes are summarized.

## 2. Materials and Experimental Details 

### 2.1. Materials

The PVDF piezoelectric films were purchased from the commercial company (Shaanxi Huiyuan Technology Co., Ltd., Xianyang, China). Their PVDF films are of good quality and are widely used in smart devices. The thickness of the thin film is 100 μm. The polarization mode of PVDF thin films was corona polarization. The PVDF film passed between two electrodes with high voltage, which put the dipole chain orientation alignment in the direction of the electric field. Then, the PVDF thin film completes the polarization and exhibits the piezoelectric effect [44]. The material properties of the PVDF thin films provided by manufacturer are listed in Table 1. The hardness and elastic modulus of the PVDF thin films were measured by nanoindentation technology (TI980 TriboIndenter, Bruke Nano. Inc., Penang, Malaysia). The hardness was about 92.6 MPa. The initial surface roughness of PVDF films was measured as *Ra* = 0.072 μm by 3D optical profiler. Note that the PVDF samples used in tests are cut from the same PVDF film, and the surface roughness can be considered a fixed value. This can minimize the effect of the initial roughness on the experimental results. The double-edge notched-tension method developed by Iglesias Montes et al. [45] was used to obtain the stress intensity factor K_IC_ in the experiments. The value of K_IC_ was obtained as $13.04\pm 0.49\mathrm{MPa}\sqrt{m}$.

### 2.2. Preparation of Test Specimens

**Figure 1:**
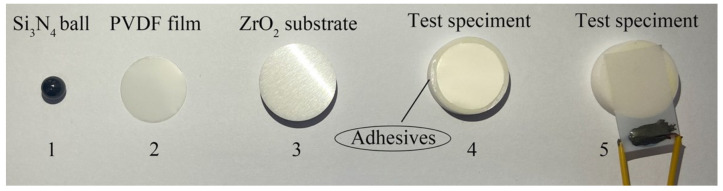
The ball and test specimen: 1—ball, 2—PVDF film, 3—ZrO_2_ substrate, 4—test specimen without the voltage, 5—test specimen with the voltage.

The ball used in the fretting test was made of Si3N4 with Poisson’s ratio of 0.26, a surface roughness of *Ra* = 0.02 μm, Young’s modulus 300 GPa, and a mass density of 3230 ${\mathrm{kg}/m}^{3}$. The diameter of the ball was 6 mm. The test specimen mainly included two parts: the PVDF film (a diameter of 16 mm and a thickness of 100 μm) and the ZrO_2_ ceramic substrate (a diameter of 20 mm and a thickness of 5 mm). The density and Young’s modulus of the ZrO_2_ ceramic are 6080 ${\mathrm{kg}/m}^{3}$ and 190 GPa, respectively. A/B industrial-grade epoxy adhesive was used to bond ZrO_2_ and the PVDF thin film. After curing for 24 h, the lap shear strength of the weather adhesive was about 23 MPa. The ball and test sample are shown in Figure 1. As for the test sample with the voltage, the connection between the wires and PVDF film was accomplished by the epoxy conductive adhesive.

### 2.3. Fretting Wear Test

**Figure 2:**
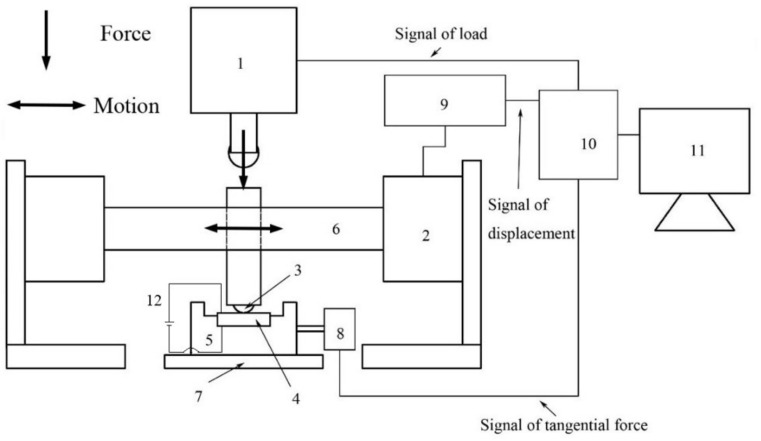
Schematic diagram of fretting wear test setup: 1—pressure sensor, 2—electromagnetic actuator, 3—sphere sample, 4—plate sample, 5—base, 6—connecting rod, 7—screw module, 8—piezo board, 9—voice coil board, 10—control unit, 11—computer, 12—constant voltage power.

The fretting wear tests were performed on the multi-function tribometer (MFT-3000, RTEC-Instruments, Inc. San Jose, CA, USA) using a ball-on-disk sliding geometry. The schematic diagram of the fretting wear test setup is shown in Figure 2. The reciprocating displacement is triggered by electromagnetic actuator, causing the motion of the connecting rod and spherical punch. The normal force is applied by the pressure sensor, which provides the data to the computer [46]. The tests were performed under normal conditions with 40 ± 5% relative humidity and 20 ± 1 °C temperature. Before the test, the balls and test specimens were thoroughly cleaned with the ethanol for 8 min in the ultrasonic cleaner, and then dried by the blower.

The experimental parameters were set as follows: imposed displacement amplitude *D* from ±2.5 μm to ±40 μm, normal force $F_{n}$ from 15 N to 50 N, frequency f from 5 HZ to 50 Hz, number of cycles *M* from 1 to 10^6^ cycles and applied voltage $V_{c}$ from 0 V to 9 V. The experimental duration was dependent on the number of cycles *M*. $F_{t}$ is the tangential fretting force, which can be recorded by sensors during the test with MFT-3000. The data on the tangential force $F_{t}$ versus the relative tangential displacement *d* were recorded by MFT-3000 as 50 points at each cycle. The friction coefficient μ was recorded as $\mu =F_{tavg}/F_{n}$ by the apparatus. The test was repeated 2–3 times for each setting condition, and only representative results are presented when the behavior was observed to be consistent. 

### 2.4. Characterizations

After the test, the scanning electron microscope (SEM, HITACHI S-4800, Hitachi, Ltd. Tokyo, Japan) was used to determine the micrographs of fretting surfaces. The specimen was sputtered with a thin layer (1–2 nm) of gold under vacuum. The 3D optical profiler (ZegageTM Plus, Zygo corp., Middlefield, CT, USA) was applied to characterize the surface profile and calculate the wear volume ΔV of PVDF thin films. To some extent, it was difficult to measure the absolute wear volume because of the creep and material transfer in the process of the fretting test. So, the specific wear rate $K_{s}\left({\mathrm{mm}}^{3}/N\cdot m\right)$ was introduced to express the relative change in the wear volume. It can be written as [47]

(1) $$K_{s}=\frac{\Delta V}{F_{n}L},$$

where $F_{n}$ is the normal force, and L is the total running distance.

**Figure 3:**
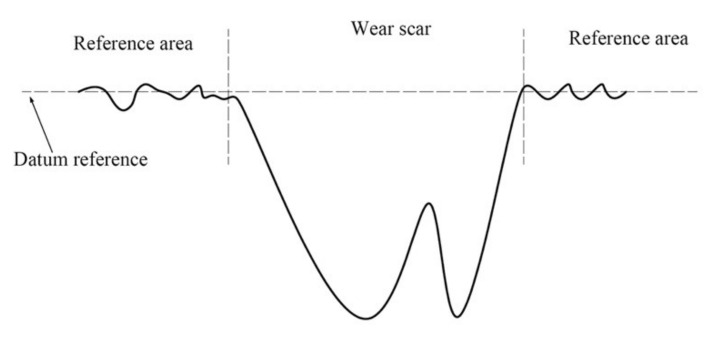
Schematic map for evaluating the wear volume.

The schematic map for determining the wear volume is shown in Figure 3. The detailed procedure is given as:

1. Scan the surface with a white light interference profilometer and obtain the information of the wear scar.
2. Select the area outside the wear scar as the reference area. The average height of all points in the reference area is the height of the datum reference.
3. Then, the software of the 3D optical profiler will compute the wear volume value for all the points under datum reference.

## 3. Results and Discussion

In this section, the fretting wear behavior of PVDF piezoelectric thin films was tested by using the multi-function tribometer with a ball-on-disk sliding geometry. The friction curves were used for distinguishing the different fretting regions. The corresponding surface damage modes were identified with the application of 3D optical profiler and SEM. The wear volume and specific wear rate were calculated to characterize the durability of the PVDF thin films under the setting fretting condition. 

We would like to explain why we only choose a kind of piezoelectric thin film in our experimental study. So far, no work was reported for the fretting wear behavior of piezoelectric thin films. In order to make a detailed description of the fretting wear behavior of piezoelectric thin films, we selected PVDF thin films as an example to give a full discussion. In the analysis, the fretting wear test results under different normal force, displacement amplitude, and external voltage are summarized through the analysis of experimental data. The fretting wear mechanism is also discussed in detail. 

### 3.1. Curves of Friction

In the tangential fretting studies, the tangential force $F_{t}$ vs. the relative displacement *d* curves ($F_{t}-d$ curves) as a function of the number of cycle M ($F_{t}-d-M$ curves) are very important. On the one hand, they can reflect the interfacial contact state during the fretting test, including the partial slip region, mixed region, and gross slip region [20,21,48]. On the other hand, the $F_{t}-d$ curve can represent the dissipated energy of a fretting cycle from which the dissipated energy can be calculated [49].

**Figure 4:**
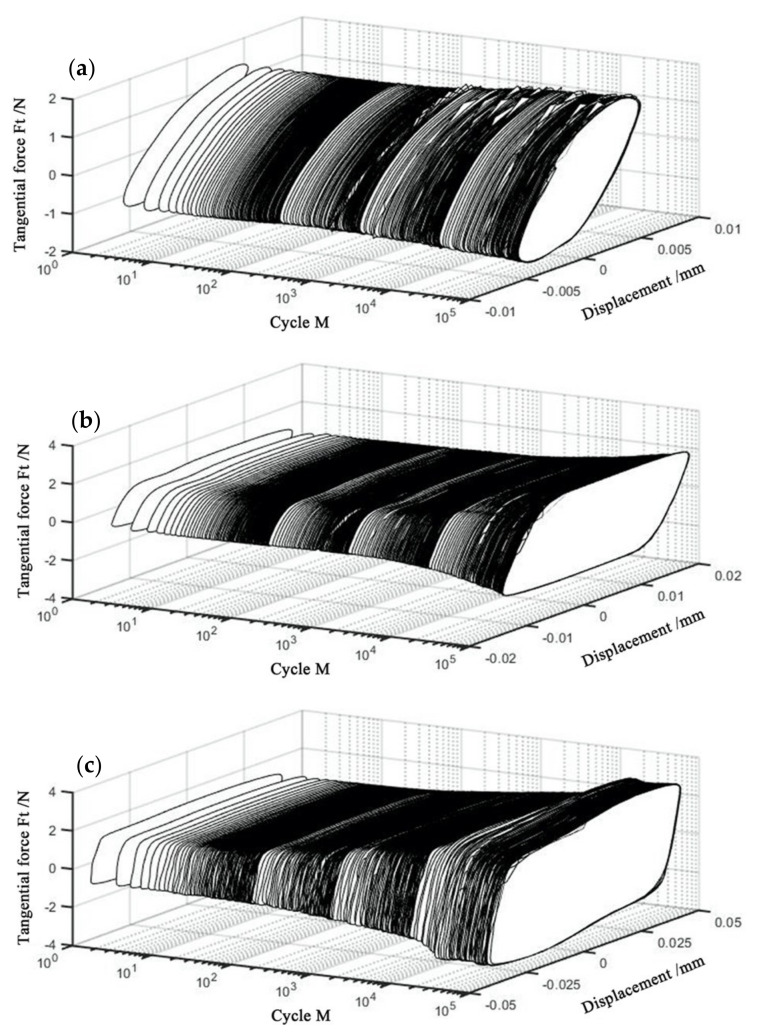
Curves of PVDF thin films with *F_n_* = 15 N, ƒ = 15 Hz, and *V_c_* = 0 V; (**a**) D = ±5 μm, (**b**) D = ±15 μm, and (**c**) D = ±40 μm.

**Figure 5:**
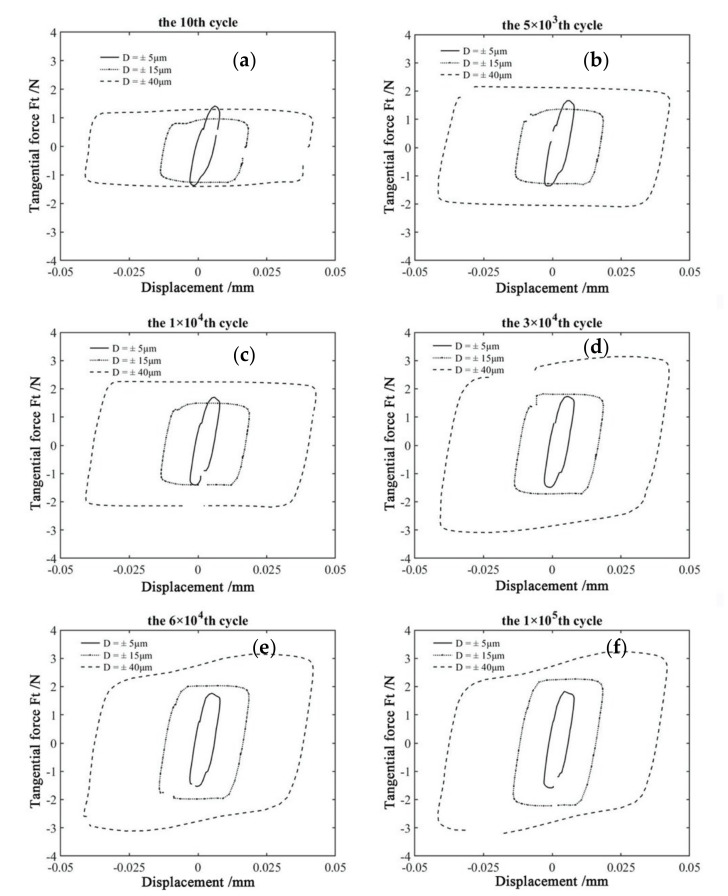
The comparison of $F_{t}-d$ curves of PVDF thin films in different number of cycles with *F_n_* = 15 N, ƒ = 15 Hz, and *V_c_* = 0 V (**a**) 10th cycle, (**b**) 5 × 10^3^ th, (**c**) 1 × 10^4^ th, (**d**) 3 × 10^4^ th, (**e**) 6 × 10^4^ th, and (**f**) 1 × 10^5^ th.

Figure 4 shows $F_{t}-d-M$ curves of PVDF thin films at various displacement amplitudes with the normal force *F_n_* = 15 N, frequency ƒ = 15 Hz, and voltage *V_c_* = 0 V. The corresponding $F_{t}-d$ curves are given in Figure 5 for the given M. It can be seen that all $F_{t}-d$ curves appear in the oval-like shape when the displacement amplitude D = ±5 μm. However, the relative motion is adjusted by the elastoplastic deformation when the displacement amplitude increases to ±15 μm and ±40 μm, and all $F_{t}-d$ curves appear in the quasi-parallelogram shape. It is indicated that the partial slip region is formed at the small displacement amplitude (D = ±5 μm), and the fretting running state is transformed from the partial slip region to the gross slip region at a great displacement amplitude (±15 μm and ±40 μm). The reason for this transition is that a great displacement amplitude will lead to large amounts of material exfoliate and debris formation during the experimental process. Please refer to the SEM images in Section 3.2 for details.

**Figure 6:**
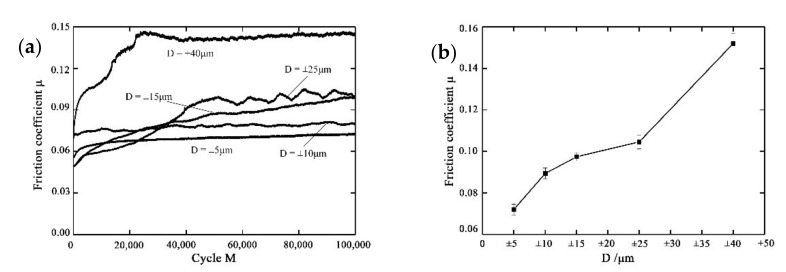
The effect of the displacement amplitude on the friction coefficient of PVDF thin films with *F_n_* = 15 N, ƒ = 15 Hz, and *V_c_* = 0 V: (**a**) variation with the number of cycles, (**b**) error bar.

Figure 6 presents the effect of the displacement amplitude D on the friction coefficient μ of PVDF thin films with *F_n_* = 15 N, ƒ = 15 Hz, and *V_c_* = 0 V. Figure 6b gives the friction coefficient at different displacement amplitudes with the error bar. The error bars are based on the repetitive measurement. Before the steady state is reached, the friction coefficient increases with the increase in *M*. At the steady state, the friction coefficient is smallest for D = ±5 μm, while it is greatest for D = ±40 μm. With the increase in the *D* from ±5 μm to ±40 μm, the friction coefficient increases from 0.07 to 0.15. In addition, there is an evident fluctuating tendency of the friction coefficient at D = ±25 μm. The reason is that an unstable contact state is formed between the two contact bodies. This unstable state is related to the fretting damage at the contact region.

**Figure 7:**
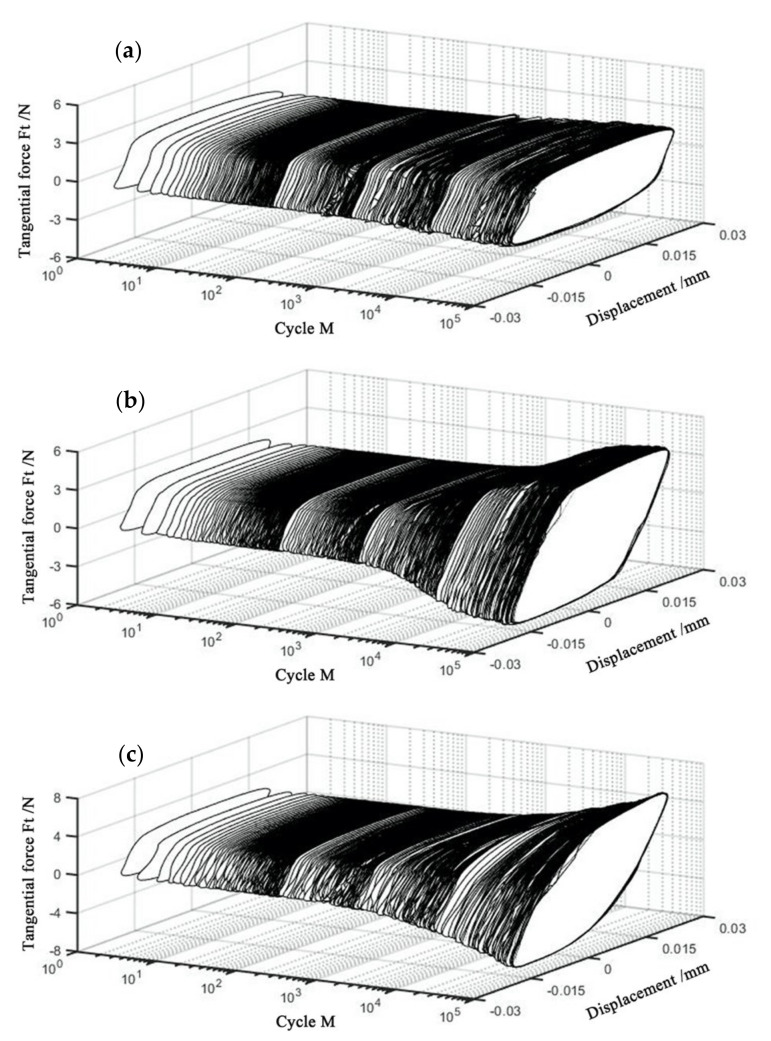
Curves of PVDF thin films with D = ±20 μm, ƒ = 15 Hz, and *V_c_* = 0 V: (**a**) *F_n_* = 15 N, (**b**) *F_n_* = 35 N, and (**c**) *F_n_* = 50 N.

**Figure 8:**
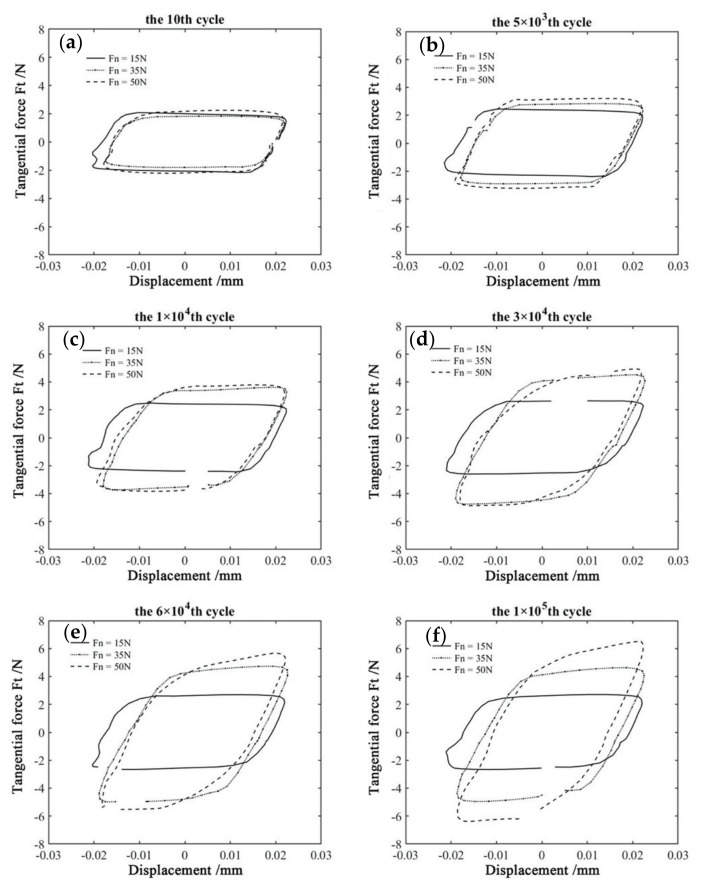
The comparison of $F_{t}-d$ curves of PVDF thin films in different number of cycle with D = ±20 μm, ƒ = 15 Hz, and *V_c_* = 0 V: (**a**) 10th, (**b**) 5 × 10^3^ th, (**c**) 1 × 10^4^ th, (**d**) 3 × 10^4^ th, (**e**) 6 × 10^4^ th, and (**f**) 1 × 10^5^ th.

Figure 7 shows the $F_{t}-d-M$ curves of the PVDF thin films at various normal forces with D = ±20 μm, ƒ = 15 Hz, and *V_c_* = 0 V. The corresponding $F_{t}-d$ curves at given *M* are shown in Figure 8. For *F_n_* = 15 N, 35 N and 50 N, we can see that their hysteresis loops are similar at the beginning of the fretting tests. They all appear in the quasi-parallelogram shape, and the gross slip state is formed. With the increase in *M*, the $F_{t}-d$ curves change from a quasi-parallelogram shape to an oval-like shape for *F_n_* = 50 N. The change of the shape is due to the influence of plastic deformation, and creep becomes more significant under a relatively large normal load, which can be observed in the SEM images (Please refer to Section 3.2). We can observe that the increasing tangential force and the typical friction logs of the mixed fretting region are formed during the fretting tests. Furthermore, the friction coefficient keeps increasing until the stable stage is reached.

**Figure 9:**
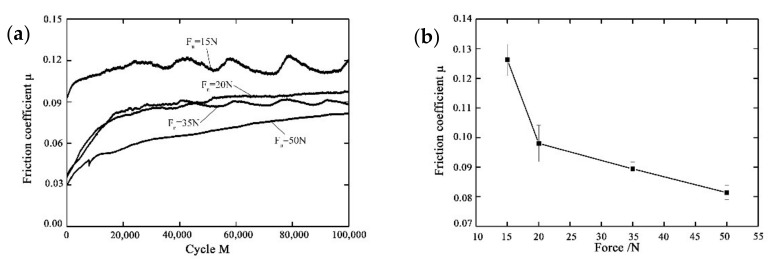
The effect of the normal force on the friction coefficient of PVDF thin films with with D = ±20 μm, ƒ = 15 Hz, and *V_c_* = 0 V: (**a**) variation with the number of cycles, (**b**) error bar.

The effect of the normal force on the friction coefficient at different displacement amplitudes with the error bars are shown in Figure 9 with D = ±20 μm, ƒ = 15 Hz, and *V_c_* = 0 V. Figure 9b gives the friction coefficient at different normal forces with the error bar. There is an initial stage (about 10^3^ cycles) at the beginning of the fretting test, then the duration of the running-in stage lasts for a while (about 2 × 10^4^ cycles). After that, the fretting test enters the relatively steady stage. The magnitude of the friction coefficient is positively related to the effective contact area and negatively related to the normal force [50]. Therefore, the fretting test at *F_n_* = 15 N has the maximum friction coefficient around 0.115, and it decreases to 0.097, 0.089, and 0.081 at *F_n_* = 20 N, *F_n_* = 35 N, and *F_n_* = 50 N, respectively. 

### 3.2. Surface Wear Topography

**Figure 10:**
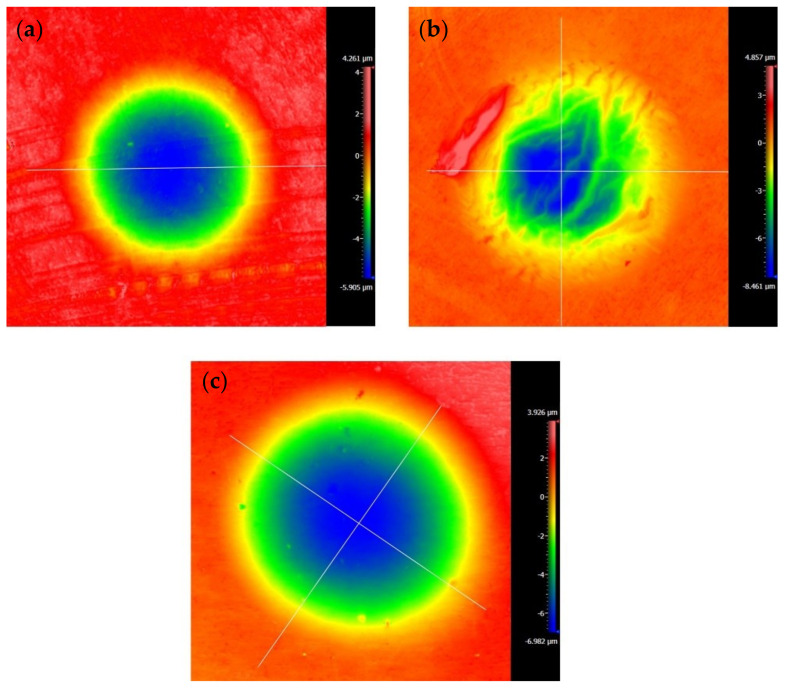
The profile of wear scars of PVDF thin films with *F_n_* = 15 N, ƒ = 15 Hz, and *V_c_* = 0 V: (**a**) D = ±5 μm, (**b**) D = ±15 μm, and (**c**) D = ±40 μm.

**Figure 11:**
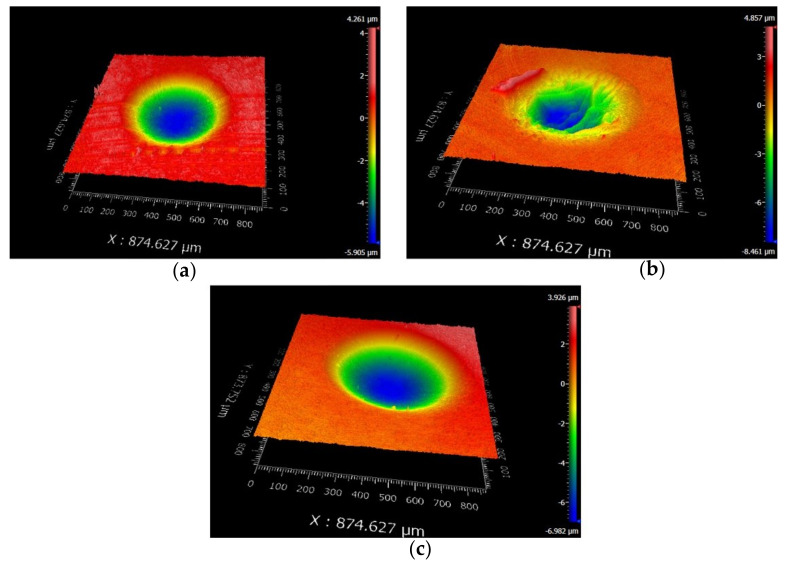
The 3D shape of PVDF thin films with *F_n_* = 15 N, ƒ = 15 Hz, and *V_c_* = 0 V: (**a**) D = ±5 μm, (**b**) D = ±15 μm, and (**c**) D = ±40 μm.

**Figure 12:**
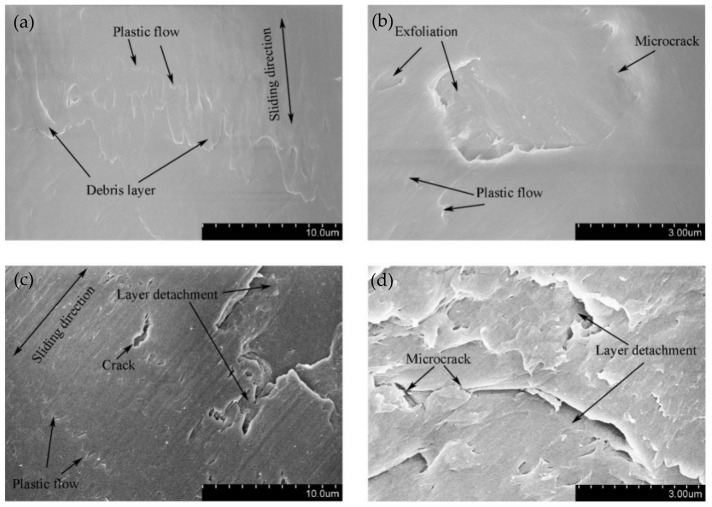
The scanning electron microscope (SEM) morphology of PVDF films in the contact region with *F_n_* = 15 N, ƒ = 15 Hz, and *V_c_* = 0 V: (**a**,**b**) D = ±15 μm and (**c**,**d**) D = ±40 μm.

**Figure 13:**
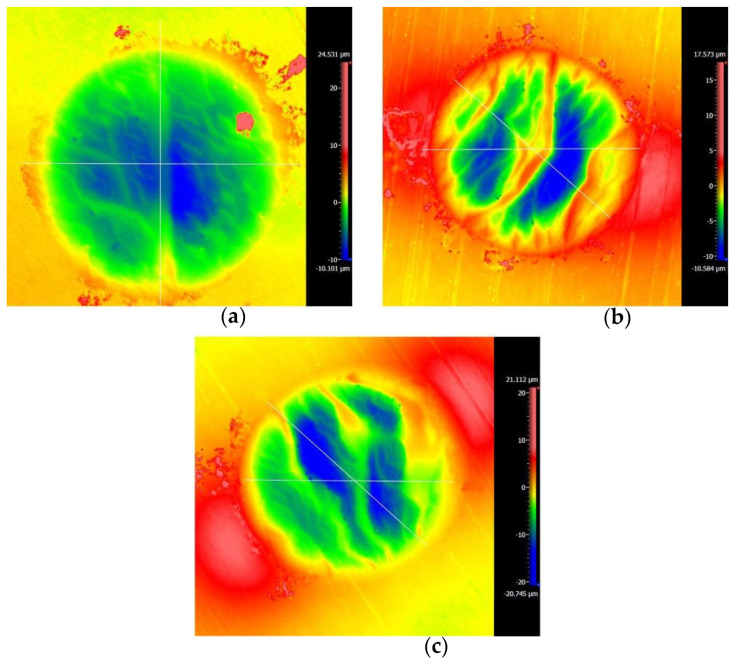
The profile of wear scars of PVDF thin films with ƒ = 15 Hz, D = ±20 μm, and *V_c_* = 0 V: (**a**) *F_n_* = 15 N, (**b**) *F_n_* = 35 N, (**c**) *F_n_* = 50 N.

**Figure 14:**
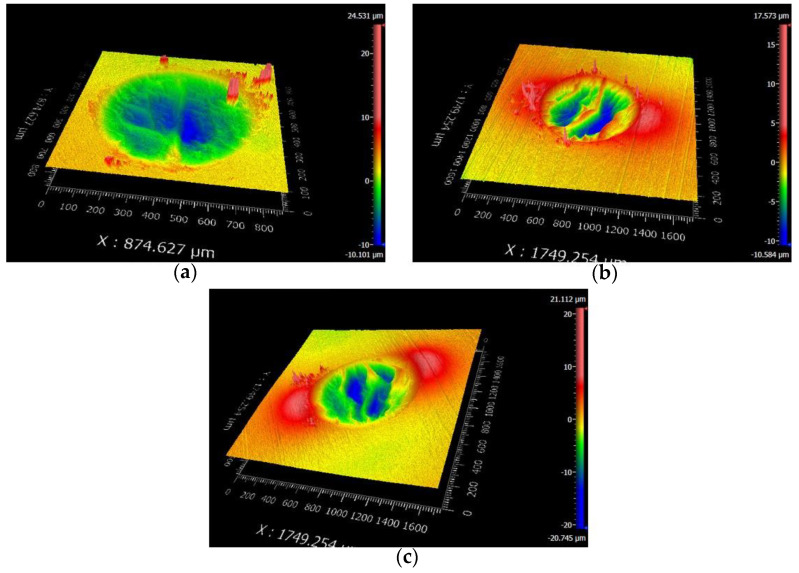
The 3D shape of PVDF thin films with ƒ = 15 Hz, D = ±20 μm, and *V_c_* = 0 V: (**a**) *F_n_* = 15 N, (**b**) *F_n_* = 35 N, (**c**) *F_n_* = 50 N.

**Figure 15:**
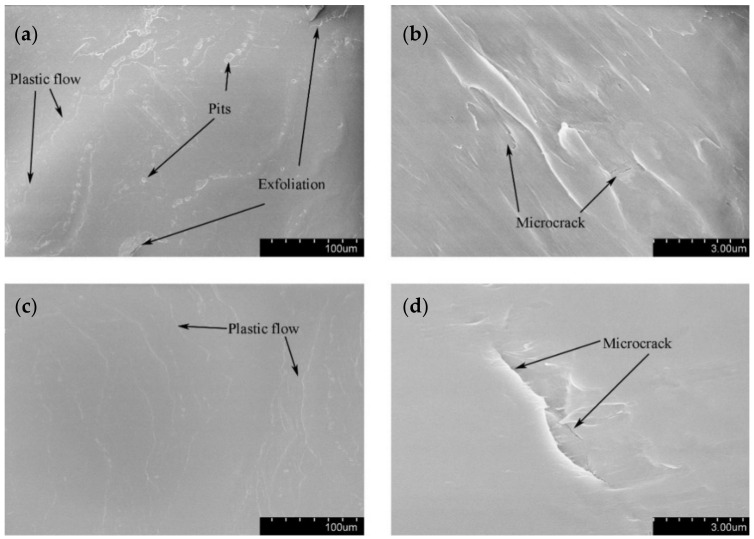
The SEM morphology of PVDF films in the contact region with ƒ = 15 Hz, D = ±20 μm, and *V_c_* = 0 V: (**a**,**b**) *F_n_* = 15 N and (**c**,**d**) *F_n_* = 50 N.

**Figure 16:**
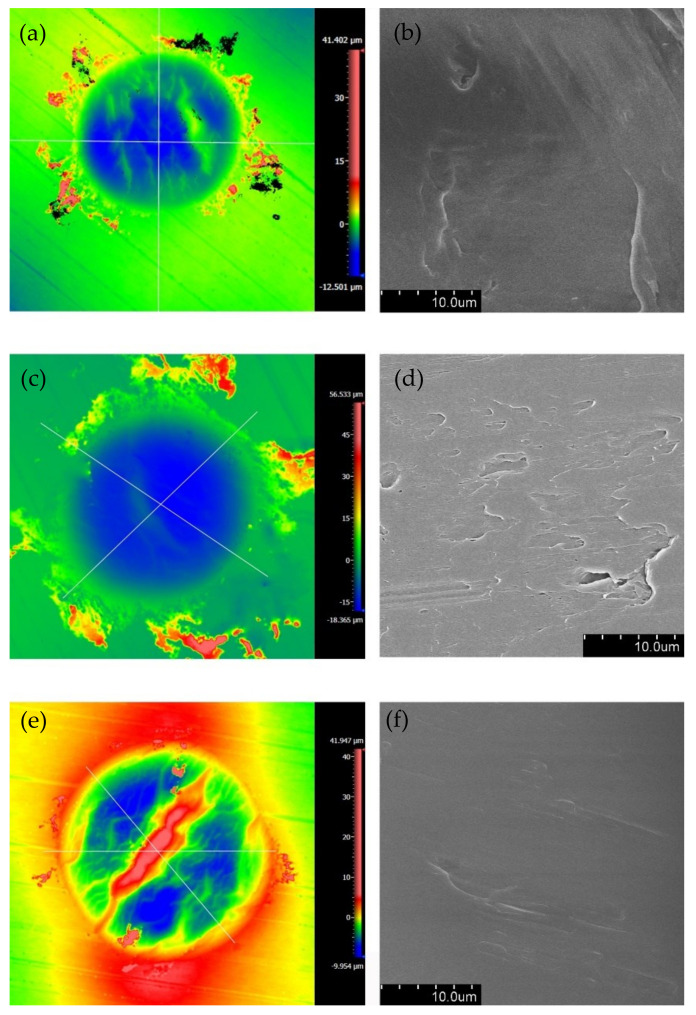
The 2D surface topographies and SEM topographies of PVDF thin films with ƒ = 30 Hz and *V_c_* = 0 V: (**a**,**b**) topographies with *F_n_* = 15 N and D = ±20 μm, (**c**,**d**) topographies with *F_n_* = 15 N and D = ±40 μm, (**e**,**f**) topographies with *F_n_* = 50 N and D = ±20 μm.

To understand how the wear mechanism works, it is important to study the wear scars at the contact surface. Thus, the study of the worn surface under different fretting conditions was carried out in Figure 10, Figure 11, Figure 12, Figure 13, Figure 14, Figure 15, Figure 16 by using a 3D optical profiler and SEM.

Figure 10 and Figure 11 are the 2D and 3D surface topography for different displacement amplitudes with *F_n_* = 15 N, ƒ = 15 Hz, and *V_c_* = 0 V, respectively. In Figure 11, “874.627 μm” is the size of the surface topography at the x-direction. On the right-hand side, the color and number represent the relative height of the wear surface. The observation of the contour of worn surfaces shows that the smaller/larger displacement amplitudes (±5 μm/±40 μm) lead to a smoother contour line. However, for the moderate displacement amplitudes, the center of the contact region forms the wrinkle and the contour of the wear scar fluctuates. The existence of a convex contour at the edge of the contact region indicates that the wear debris piles up. This phenomenon is quite remarkable for D = ±15 μm. The reason can be attributed to the change of the contact region caused by the varying displacement amplitude. For a smaller displacement amplitude (D = ±5 μm), the partial slip region is formed as shown in Figure 4 and Figure 5. There is no relative slip at the contact region, and slight abrasive wear is formed at the contact surface; the contact basically conforms to the Mindlin’s elastic contact theory. When the displacement amplitude increases to D = ±15 μm, the fretting loops change to a quasi-parallelogram shape. Moreover, the relative displacement increases, and surface damage and elastic-plastic deformation are intensified at the contact surface. When the displacement amplitude further increases to D = ±40 μm, the slip region is formed, and the main damage at the contact region is delamination and exfoliation.

The SEM topography of the wear scar is shown in Figure 12 for different displacement amplitudes with *F_n_* = 15 N, ƒ = 15 Hz, and *V_c_* = 0 V. The numbers (10μm or 3μm) in the figure legend represent the scale of the SEM images. For a smaller displacement amplitude (D = ±15 μm), the deformation at the contact region is dominant in the plastic deformation. We can see that the plastic debris layer is formed, and the exfoliations and microcracks occur at a very small scale. For a relatively greater displacement amplitude (D = ±40 μm), the large-scale layer detachment and apparent cracks are formed (Figure 12c), the surface is relatively rough, and the friction coefficient increases. Obviously, the increase in the displacement amplitude will lead to the increase in the wear volume of the PVDF thin films. Because the surface profile fluctuates sharply and the surface wrinkles are deepened at the contact region, the fretting wear mechanism of the PVDF thin film is complex and can be considered a self-organized process containing a variety of interrelated mechanisms of friction and deformation [51].

Figure 13 and Figure 14 are the 2D and 3D surface topography for different normal forces with ƒ = 15 Hz, D = ±20 μm, and *V_c_* = 0 V, respectively. It is apparent that the profiles of contact surface are significantly affected by the normal force. With the increase in the normal force from 15 N to 50 N, we can see that the distance between the material surface and the lowest point of the contour increases from 9 μm to 15 μm. Additionally, more pronounced plastic deformation occurs, the surface profile fluctuates sharply, and the surface wrinkles are deepened at the contact region. This phenomenon can be used to explain the results in Figure 7 and Figure 8. We can observe in Figure 7 and Figure 8 that the increase in the normal force leads to the change of $F_{t}-d$ curves from a quasi-parallelogram shape to an oval-like shape after 10^4^ cycles. The reason is that the influence of the plastic deformation is intensified for the large normal force.

The SEM topography of the wear scar is shown in Figure 15 for different normal forces with ƒ = 15 Hz, D = ±20 μm, and *V_c_* = 0 V. When the normal force *F_n_* = 15 N, the evident pit and exfoliation are observed in Figure 15a. When *F_n_* = 50 N, the plastic deformation and creep which result from squeezing stress dominate the wear of the contact surface. The apparent transverse ridges are formed along the fretting direction. 

The 2D surface topographies and SEM topographies of the PVDF thin films with ƒ = 30 Hz and *V_c_* = 0 V are shown in Figure 16. Compared to Figure 10, Figure 11, Figure 12, Figure 13, Figure 14 and Figure 15 with ƒ = 15 Hz, we can observe a similar phenomenon in Figure 16 with ƒ = 30 Hz:

1. The plastic deformation causes surface wrinkles when D = ±20 μm, and the increase in displacement amplitude (D = ±40 μm) makes the surface wrinkles disappear
2. The wear depth increases with the increase in the displacement amplitude
3. The increase in normal force makes the plastic deformation and creeps more distinct. The same phenomenon was also observed by Wang et al. [52]. Referring to Maruschaket et al. [53], the possible causes for fretting wear damage are summarized in Table 2.

### 3.3. Wear Volume and Specific Wear Rate

In the present tests, the wear volumes of PVDF thin films were measured after each test by using the 3D optical profiler. Furthermore, to ensure the reliability of results and reduce experimental error, the wear volume of each sample was tested three times. Error bars were employed to analyze the wear volume and specific wear rate. The specific wear rate is calculated from Equation (1).

**Figure 17:**
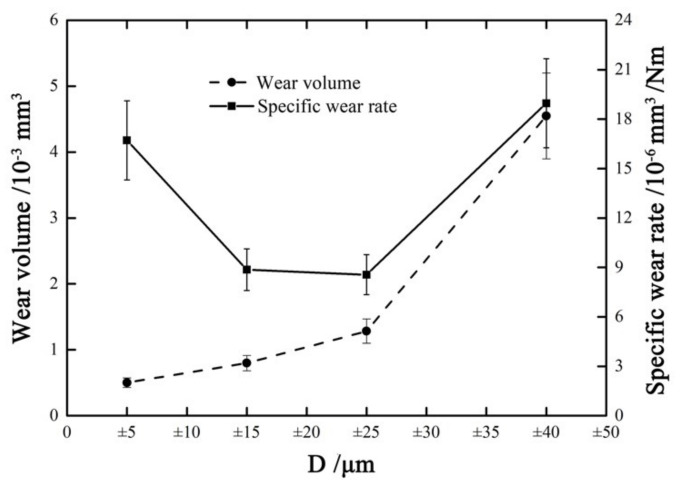
Wear volume and specific wear rate versus displacement amplitude with *F_n_* = 15 N, ƒ = 15 Hz, and *V_c_* = 0 V.

Figure 17 shows the wear volume and specific wear rate vs. displacement amplitude curves with *F_n_* = 15 N, ƒ = 15 Hz, and *V_c_* = 0 V. When the displacement amplitude *D* increases from ±5 μm to ±40 μm, the wear volume increases from 0.5 × 10^−3^ mm^3^ to 4.5 × 10^−3^ mm^3^. However, the evolution of the specific wear rate has a different tendency compared with the wear volume. The specific wear rate decreases at first and then increases with the increase in *D*. The lowest specific wear rate (about 9 × 10^−9^ mm^3^/Nm) appears at D = ±25 μm. The reason is that when the contact region is running in partial slip region or mixed region, the plastic deformation and creep that create a large plastic zone in the center of the contact zone, and the presence of the plastic zone can hinder crack growth and aggravate damage [52,53]. To some extent, the increase in the plastic zone contributes to the strain energy absorption and increases the material toughness in the plastic zone. Therefore, the specific wear rate will tend to decrease first with the increase in displacement amplitude. However, the wear volume always shows an increasing trend with the increase in displacement amplitude.

**Figure 18:**
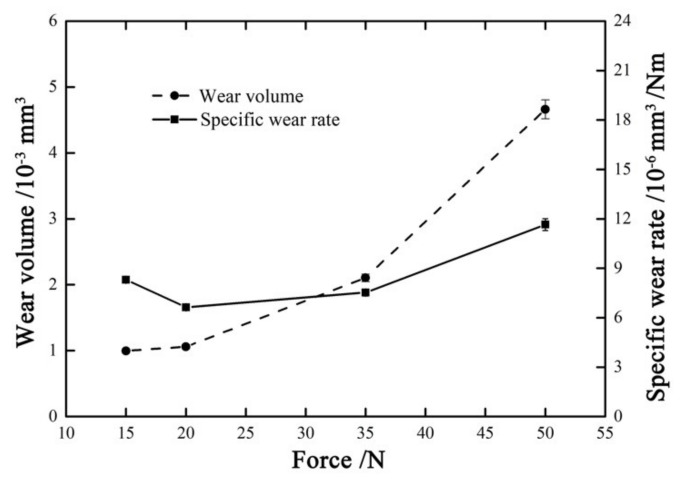
Wear volume and specific wear rate versus normal force with ƒ = 15 Hz, D = ±20 μm, and *V_c_* = 0 V.

Figure 18 shows the wear volume and specific wear rate versus normal force curves with ƒ = 15 Hz, D = ±20 μm, and *V_c_* = 0 V. With the increase in the normal force from 15 N to 50 N, the wear volume increases, while the specific wear rate decreases first and then increases. The minimum wear volume about 1 × 10^−3^ mm^3^ appears at *F_n_* = 15 N, and the maximum wear volume about 4.6 × 10^−3^ mm^3^ appears at *F_n_* = 50 N. It is obvious that the increase in the normal force will lead to more wear volume. 

### 3.4. Fretting Wear under the Applied Voltage

**Figure 19:**
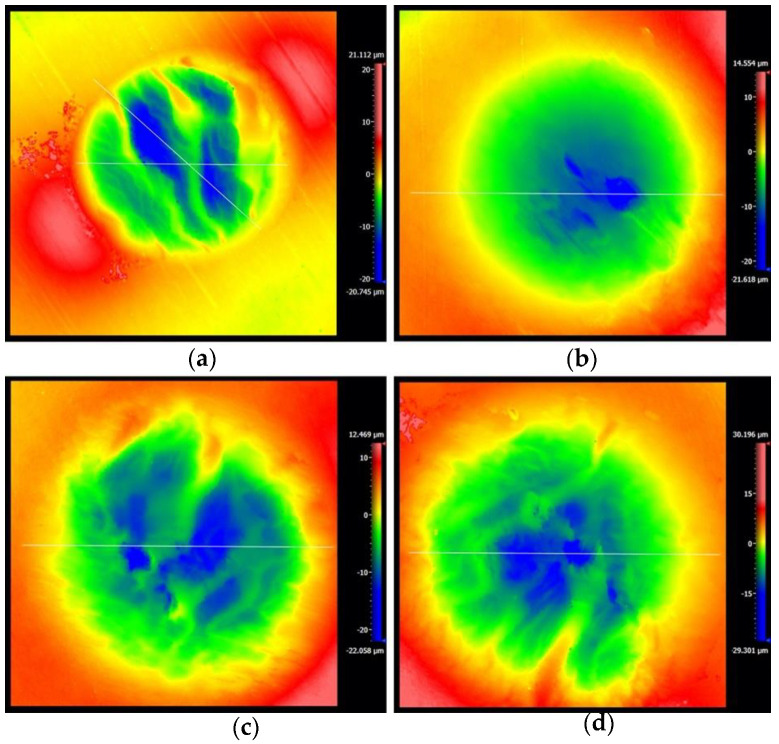
The 3D shape of PVDF thin films with *F_n_* = 50 N, D = ±20 μm and ƒ = 15 Hz: (**a**) 0 V, (**b**) 3 V, (**c**) 6 V, and (**d**) 9 V.

**Figure 20:**
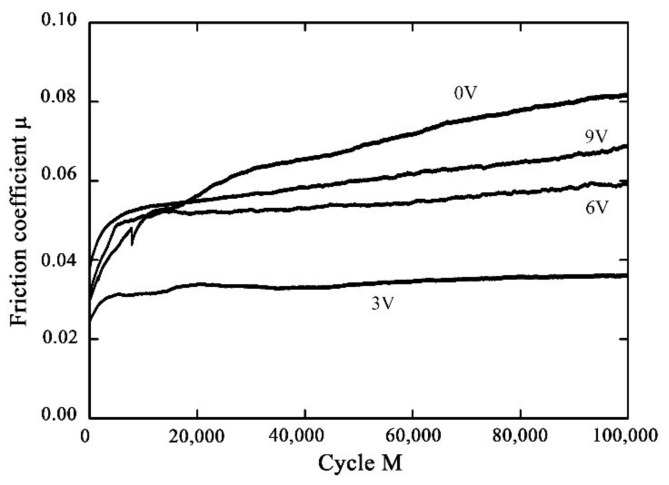
The effect of the voltage on the friction coefficient curve of PVDF thin films with *F_n_* = 50 N, D = ±20 μm and ƒ = 15 Hz.

In this sub-section, the effect of the applied voltage on the fretting wear behavior of the PVDF thin film is considered. Figure 19 shows the surface topography for different voltages with *F_n_* = 50 N, D = ±20 μm and ƒ = 15 Hz. When the voltage is applied along the direction of polarization, the piezoelectric materials will elongate along the polarization direction because of the inverse piezoelectric effect, and in turn they will affect the contact surface in the process of fretting wear [54]. The existence of voltage makes the surface of the piezoelectric material relatively smooth. With the voltage increase from 0 V to 9 V, the surface profile of the PVDF films is smoothest at a voltage of 3 V, and plastic deformation is rare, which leads to the reduction of the friction coefficient. This phenomenon is also reflected in the friction coefficient curve in Figure 20. There is a minimum friction coefficient (around 0.035) when the voltage *V_c_* = 3 V, and then the friction coefficient increases with the increase in voltage from 3 V to 9 V. It is worth mentioning that when no voltage is applied during the fretting process, the stability phase of the fretting process has the maximum friction coefficient (around 0.08).

**Figure 21:**
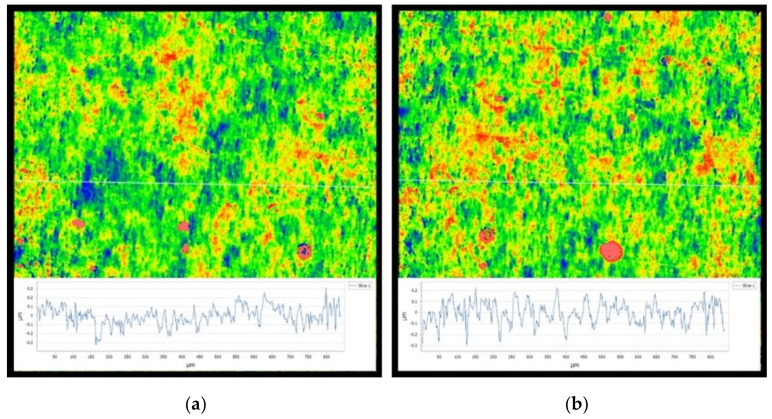
The surface topography of the PVDF thin film before the test: (**a**) 0 V and (**b**) 3 V.

Figure 21 shows the surface topography of the PVDF thin film before the test. The surface height characteristic parameters (*Ra*, *Rz*) are recorded. The surface height characteristic parameters of PVDF film are Ra=0.072μm and Rz=1.203μm when *V_c_* = 3V, and they are Ra=0.079μm and Rz=1.596μm when *V_c_* = 0 V. Clearly, the parameters (*Ra*, *Rz*) of *V_c_* = 3 V are smaller than those of *V_c_* = 0 V. This result can be used to explain why the friction coefficient of *V_c_* = 3 V is smaller than that of *V_c_* = 0 V.

**Figure 22:**
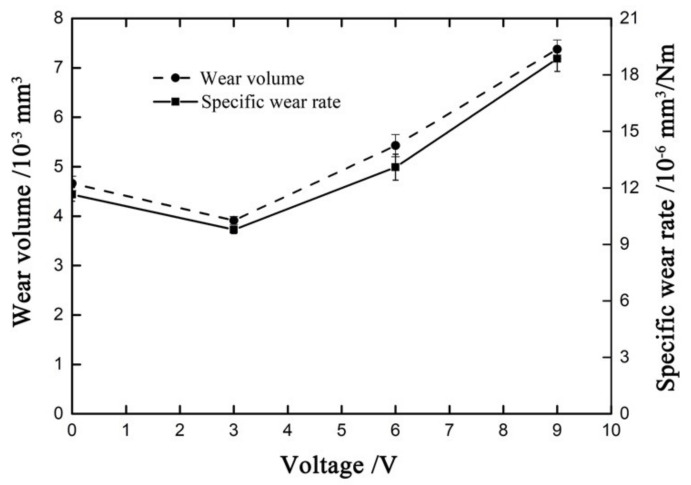
Wear volume and specific wear rate versus voltage with *F_n_* = 50 N, D = ±20 μm and ƒ = 15 Hz.

Figure 22 shows the wear volume and specific wear rate versus voltage curves with *F_n_* = 50 N, D = ±20 μm and ƒ = 15 Hz. The wear volume and specific wear rate show the same trend. With the increase in voltage, both wear volume and specific wear rate first increase and then decrease and reach the minimum value at *V_c_* = 3 V, where the wear volume is 4.65 × 10^−3^ mm^3^ and the specific wear rate is 11.65 × 10^−6^ mm^3^/Nm. 

## 4. Conclusions 

This paper focused on the fretting wear behavior of PVDF thin films in air condition. The friction logs were used to determine the contact state of the thin PVDF thin film during the fretting test. The 3D topography instrument and SEM were used to measure the surface morphology and wear volume. From the experimental data and morphological characterization, the following conclusions are drawn:

1. The friction coefficient of the PVDF thin films shows an increasing trend from 0.07 to 0.144 with the increase in displacement amplitude, and shows a decreasing trend from 0.115 to 0.081 with the increase in applied normal force under the setting condition.
2. The increase in displacement amplitude leads to the change of the contact region from the partial slip region to the gross slip region. However, the increase in normal force results in the change of the contact region from the gross slip region to the mixed region.
3. The plastic deformation and creep cause surface wrinkles, and the increase in displacement amplitude will reduce or even eliminate the surface wrinkles.
4. The wrinkles in the mixed region are more obvious than in the partial/gross slip regions.
5. With the increase in the displacement amplitude (from ±5 μm to ±40 μm) and normal force (from 15 N to 50 N), the wear volume increases, while the specific wear rate decreases first and then increases.
6. The fretting wear mechanism of the PVDF thin film is complex and can be considered as a self-organized process, containing a variety of interrelated mechanisms of friction and deformation
7. With the increase in voltage, both wear volume and specific wear rate first increase and then decrease, and reach the minimum value at *V_c_* = 3 V.

This paper is a preliminary study on the fretting wear of piezoelectric thin films. It still has some limitations, which will be solved in our future works. Some precise directions related to the present works include:

1. To consider different piezoelectric thin films (PVF, PZT, ZnS, ZnO, etc.) and compare their fretting wear behavior;
2. To discuss the effect of the external applied voltage and prestress;
3. To consider other fretting modes, such as rotational fretting, radial fretting, torsional fretting, and composite fretting;
4. To carry out the finite element simulation on the fretting wear of piezoelectric thin films and compare with the experimental results.

## Figures and Tables

**Table 1:** Material properties of the polyvinylidene fluoride (PVDF) piezoelectric thin film.

Properties	Units	Value
Density, ρ	kg/m^3^	1.78 × 10^3^
Young’s modulus, E	GPa	1
Poisson’s ratio	-	0.35
*d*_33_ constant	10^−12^ C/N	±21
Yield Strength	10^6^ N/m^2^	45–55
Tensile Strength at Break	MPa	35–50
Breaking elongation	-	20–50%

**Table 2:** Damage type and the causes for fretting wear.

Fretting Region	Damage Type	Possible Causes
The partial slip region	Abrasive wear, plastic flow	The contact basically conforms to the Mindlin’s elastic contact theory
The mixed region	Creeps, wrinkles, plastic deformation, reduced wear rate	The generation of a large plastic zone obtunds the crack growth and enhances the toughness of contact region.
The slip region	Layer detachment, crack, wear speeding up	The crack propagation causes micro-defects and material loss.

## Data Availability

All data, models, or code generated or used during the study are available from the corresponding author by request.
